# The Effect of miRNA-Modified Exosomes in Animal Models of Spinal Cord Injury: A meta-Analysis

**DOI:** 10.3389/fbioe.2021.819651

**Published:** 2022-01-06

**Authors:** Mengdie Hu, Zhidong Cao, Dianming Jiang

**Affiliations:** ^1^ Department of Orthopedics, The Affiliated Central Hospital, Chongqing University, Chongqing, China; ^2^ Department of Orthopedics, The Third Affiliated Hospital, Chongqing Medical University, Chongqing, China

**Keywords:** exosomes, animal models, spinal cord injury, miRNA, meta-analysis

## Abstract

**Background:** Spinal cord injury (SCI) is currently not completely curable. Exosomes have been widely used in preclinical studies of spinal cord injury. Here, in this meta-analysis, we focused on evaluating the overall efficacy of therapies based on miRNA-modified exosomes on functional recovery in animal models of SCI.

**Methods:** PubMed, embase and Web of Science library databases were searched. Relevant literature was included, and the random effects model was used to assess the overall effect of the intervention, with outcomes expressed as SMD. The primary outcome included motor function scores. Risk of bias (ROB) was assessed using the ROB tool of the Systematic Review Centre for Laboratory Animal Experimentation (SYRCLE). R version 4.1.1software and Review Manager software were used for meta-analysis.

**Results:** A total of 11 preclinical studies were included. The meta-analysis revealed that miRNA-modified exosome therapy was effective in improving motor function scores compared with exosomes alone or control therapy (standardized mean difference: 4.21; 95% confidence interval: 3.39–5.04). There was significant asymmetry in the funnel plot, and trim-and-fill analysis revealed four unpublished studies of motor scores. The quality of all included studies was evaluated with SYRCLE’s ROB tool. The SCI model, administration time and dose had an impact on the effect of the treatment.

**Conclusion:** MiRNA-modified exosomes have shown great potential in the treatment of SCI. Moreover, the efficacy of miRNA-modified exosomes was superior to that of exosomes alone.

## Introduction

Spinal cord injury (SCI) is mainly caused by traumatic events, such as accidents, sports injuries, and violence. The number of patients with disabilities caused by SCI worldwide exceeds 27 million (GBD 2016 Traumatic Brain Injury and Spinal Cord Injury Collaborators, 2019). SCI can cause motor and sensory dysfunction and reduce the quality of life of the affected individual (Diop et al., 2021). It also imparts a heavy economic burden on the family and society because of the high disability and mortality rates (GBD 2016 Traumatic Brain Injury and Spinal Cord Injury Collaborators, 2019; Sweis and Biller, 2017; Chamberlain et al., 2019). Current clinical methods such as spinal canal decompression surgery and drug therapy (Russo et al., 2020) are of limited efficacy and cannot fully restore the function of the damaged spinal cord (Ramer et al., 2014). Stem cells are widely used in the treatment of SCI (Vismara et al., 2017). However, stem cells can undergo undirected differentiation, which is influenced by the host microenvironment, after transplantation into the body (Yeng et al., 2016). Intravenous injection of stem cells may also cause a large number of stem cells to accumulate in the lung tissue, and furthermore, the application of stem cells is hindered by problems of tumorigenicity (Mohseny and Hogendoorn, 2011) and immune rejection.

Recently, a large number of studies have suggested that the therapeutic effect of stem cells may be attributed to their paracrine ability (Ratajczak et al., 2012; Ellison-Hughes and Madeddu, 2017; Tachibana et al., 2017; Pegtel and Gould, 2019). Exosomes are lipid bilayer vesicles (He et al., 2018) that can contain a large number of biologically active molecules, and are secreted by cells. Isolated exosomes encapsulate proteins, lipids and nucleic acids (Kalluri and LeBleu, 2020; Isaac et al., 2021), and can transmit information between cells and have biological effects similar to those of their cells of origin. Exosomes are small, with a diameter of only 30–150 nm, and they can easily pass through the blood–brain barrier, and exhibit low tumorigenicity. Exosomes can also be used as drug carriers for therapeutic use (He et al., 2018; Li D et al., 2018). It is reported that exosomes secreted by MSCs have anti-inflammatory and anti-apoptotic effects, and that they can promote angiogenesis and axon regeneration, similar to MSCs themselves (Wang et al., 2019; Huang et al., 2020).

MicroRNAs (miRNAs) are small non-coding RNAs that bind to the 3ʹ-untranslated region of the target messenger RNA (mRNA) to regulate gene expression (Liu and Xu, 2011). Numerous studies show that miRNAs can regulate cell proliferation and apoptosis, and promote angiogenesis, osteogenic differentiation, and bone regeneration (Hu et al., 2015; Su et al., 2015; Zhou et al., 2016). Several studies have reported the downregulation of a number of miRNAs after SCI. A study found that elevated miR-126 levels inhibit the inflammatory response, promote vascularization, and improve functional recovery after SCI(Hu et al., 2015). MiRNAs are highly unstable, and must be delivered through an effective carrier system. Exosomes have emerged as a promising vehicle for miRNA delivery to the central nervous system (Pegtel et al., 2010; Sancho-Albero et al., 2019). By overexpressing specific miRNAs in cell culture, and then collecting the exosomes, targeted therapy for refractory diseases can be performed. Therapeutic strategies based on miRNA-modified exosomes have recently emerged, and have been applied to a variety of diseases, including SCI. Exosomes have major advantages as therapeutic carriers because of their ability to cross biological barriers and their tissue tropism, as well as their ability to avoid immune system-mediated degradation (Zhang et al., 2016).

Despite the incomplete recovery, cell-free therapies have shown great potential in improving neurological function in animal models. We conducted a meta-analysis of data from studies of rodent models of SCI to evaluate the overall efficacy of miRNA-modified exosome therapies in promoting motor recovery in preclinical models of SCI.

## Methods

We registered this study on PROSPERO before starting the review (CRD2021255307). The protocol was established according to the Preferred Reporting Items for Systematic Review and Meta-Analysis guidelines (Moher et al., 2009) (Supplementary Table S1).

### Sources and Search Strategy

PubMed, embase and Web of Science were searched for original preclinical studies on the effects of exosomes and miRNAs on SCI published before June 2021 (Supplementary Table S2). The search strategy was as follows: the theme words (“Spinal Cord Injuries and MicroRNAs and Exosome”) and all free terms words. Literature published in English was included. References of selected articles were further screened for additional citations. The last search was performed on 8 June 2021.

### Literature Selection

All studies were stored as bibliographic references in EndNote X9.1, and two authors (Meng-Die Hu and Zhi-Dong Cao) independently selected them based on the inclusion criteria. Studies were included in this meta-analysis when they met all of the following criteria: (1) original *in vivo* animal studies using a SCI model; (2) overexpression of a single miRNA by transfection, one or more interventions at any dose; (3) exosomes or control; (4) controlled experimental studies; (5) publications written in English only. The following were excluded: (1) *in vitro* studies, not a SCI study; (2) non-single microRNAs; (3) absence of evaluation of motor function; (4) uncontrolled experimental studies; (5) non-English publication; (6) no relevant outcomes reported.

### Data Extraction

The following data extraction from the included studies was conducted independently by two authors (Meng-Die Hu and Zhi-Dong Cao). Discrepancies between them were resolved by a third reviewer (Dian-Ming Jiang). The following details were collected: (1) study characteristics: authors, year of publication, and study location; (2) animal model: species, sex, age; (3) characteristics of the intervention and comparators: miRNA variant, dose, the timing of administration, frequency of administration, route of administration; (4) outcome measures: Basso, Beattie and Bresnahan (BBB) score or Basso Mouse Scale (BMS) score. Motor function score data at 28 days were collected.

The mean and SEM or SD were extracted from the article text whenever possible. If the results were only presented as graphs, Web Plot Digitizer was used to extract numerical values (https://automeris.io/WebPlotDigitizer/) (Sistrom and Mergo, 2000). Subsequently, the collected data were transferred manually into an Excel spreadsheet for data analysis. When some data were missing and the author could not be contacted, we excluded the corresponding research from the meta-analysis.

### Quality Assessment

The methodological quality of each eligible study was assessed independently by two authors (Meng-Die Hu and Zhi-Dong Cao) using SYRCLE’s risk of bias (ROB) tool (Hooijmans et al., 2014). The items were as follows: (1) Selection bias: random sequence, allocation concealment, baseline characteristics; (2) Detection bias: blinding study team, random housing; (3) Reporting bias: random outcome assessment, blinding outcome assessors; (4) Attrition bias: incomplete outcome data; (5) Reporting bias: selective outcome reporting; (6) Other bias.

In addition, we summarized the clinical characteristics of spinal cord injury and evaluated the included studies according to the clinical relevance of the models in the study.

### Statistical Analysis

Studies were combined and analyzed using R version 4.1.1 (RStudio, USA) and Cochrane Collaboration Review Manager (version 5.4) for all statistical data and graphs. For continuous outcomes, the mean, SD and number to calculate the standardized mean difference (SMD) were extracted. The effect size was calculated using the SMD with a 95% confidence interval (CI) for continuous outcomes. Heterogeneity was determined with Tau^2^ and I^2^ statistics (Higgins and Thompson, 2002). A random effects model was used to analyze the data when heterogeneity was significant (*p* < 0.05 or I^2^ ≥ 50%). Otherwise, the fixed effects model was used. Funnel plots were used to visualize publication bias and confirmed by Egger’s regression test. Trim-and-fill analysis (Shi and Lin, 2019) was used to estimate the number of “missing” unpublished studies and calculate an adjusted effect size accounting for publication bias. Furthermore, influential analysis and several subgroup analyses were conducted to explore sources of heterogeneity in the data, including the reporting of blinding, type of injury, and dose. Subgroup analyses were predefined in the protocol and only carried out if there were at least two independent comparisons per subgroup. A *p*-value of <0.05 was considered statistically significant.

## Results

### Article Selection Process

**FIGURE 1:**
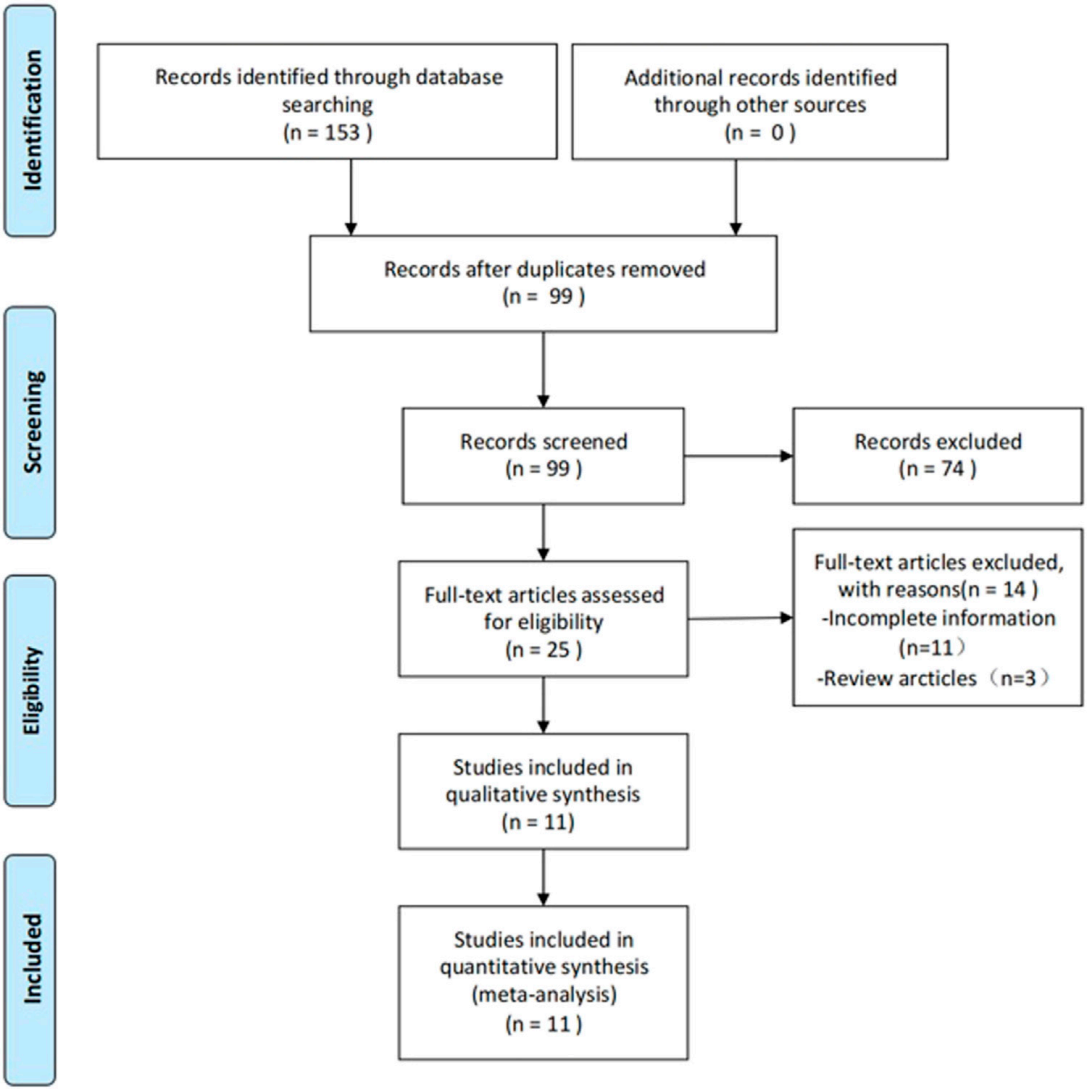
PRISMA flow chart summarized the literature search and number of included

The process of study selection is outlined in Figure 1. A total of 153 articles were identified, of which 11 articles satisfied the inclusion criteria (Ji et al., 2019; Kang et al., 2019; Yu et al., 2019; Kang et al., 2020; Liu et al., 2020; Wang et al., 2020; Jia et al., 2021; Jiang and Zhang, 2021; Zhang L. et al., 2021). After deleting duplicate articles, we excluded 74 irrelevant articles (e.g., irrelevant disease models, reviews, and conferences) by examining the title and abstract of the article. A total of 25 articles were entered into the full-text screening process. The full text of the articles was viewed according to the full inclusion criteria. Following the full-text screening process, 11 studies with incomplete information were excluded, including one study that was withdrawn, and three that were designed to investigate the development of pathophysiology in SCI rats.

### Study Characteristics and Experimental Models

The characteristics of the included studies are presented in Table 1. Most studies used rat models, and only two studies used mouse models. The thoracic spinal cord (T9-T10) was the level at which damage was assessed in all studies. Among the included studies, nine used bone marrow mesenchymal stromal cell-derived exosomes, one used human neuroepithelial stem cell-derived exosomes, and one used M2 bone marrow macrophage-derived exosomes. Seven studies used the contusion model, three used a compression model, and one study did not report a modeling method. The route of administration in all studies was intravenous. There was no great variability in the dosing strategy in the included studies. Ten studies used single-dose administration, and only one study used multiple doses. Nine studies initiated acute treatment within 1 h of injury onset. One study was initiated treatment at 24 h, and one study did not report time to treatment. Several studies reported intravenous administration of the same dose of exosomes (100 μg, 200 µg). The studies used many different miRNAs to modify exosomes, including miR-26a (Chen et al., 2021), miR-126 (Huang et al., 2020), miR-145-5p (Jiang and Zhang, 2021), miR-381 (Jia et al., 2021), miR-21 (Ji et al., 2019; Kang et al., 2019), miR-216a-5p (Liu et al., 2020), miR-421-3p (Wang et al., 2020), miR-29b (Yu et al., 2019) and miR-181c (Zhang M. et al., 2021).

**TABLE 1:** Summary of included studies.

							
Study	Species	Sex	Age	Segment	Model	Exosomes source	Type of miRNA	Dose	Route	Frequeny
chen Y 2021	SD rats	Male	6–8 weeks	T10	compression 75 g × 30 s	BMSC	miR-26a	200 μg	tail vein injection	immediately
Huang J 2020	SD rats	Male	Adult	T10	Contusion 8 g × 40 mm	BMSC	miR-126	100 μg	tail vein injection	30min
Ji W 2019	obese SD rats	Male	Adult	T10	compression 35 g × 60 s	BMSC	miR-12	100 μg	tail vein injection	24 h
Jia X 2021	SD rats	Male	6–8 weeks	T9	compression 60s	BMSC	miR-381	NA	tail vein injection	NA
Jiang Z 2021	SD rats	Male	7 weeks	T9 -T10	NA	BMSC	miR-145-5p	100 μg	tail vein injection	30min
Kang J 2019	SD rats	NA	Adult	T9 -T10	Contusion 10 g × 25 mm	BMSC	miR-21	NA	tail vein injection	immediately
Kang J 2020	SD rats	Male	Adult	T9 -T10	Contusion 10 g × 25 mm	HNESC	miR-29b	NA	tail vein injection	immediately
Liu W 2020	C57BL/6	Male	6–8 weeks	T10	Contusion 5 g × 6.5 cm	BMSC	miR-216a-5p	200 μg	tail vein injection	immediately
Wang J 2020	C57BL/6	female	8 weeks	T10	Contusion 5 g × 5 cm	BMDM	miR-421-3p	200 μg	tail vein injection	30min
Tao Y 2019	SD rats	female	NA	T10	Contusion 2 N	BMSC	miRNA-29b	100 μg	tail vein injection	1 h
Zhang M 2021	SD rats	Male	8 weeks	T10	Contusion 10 g × 12.5 mm	BMSC	miR-181c	200 μg	tail vein injection	30 min, 7 days, 14 days

SD, sprague dawley; BMSC, bone marrow mesenchymal stem cells; HNESC, human neuroepithelial stem cells; BMDM, bone marrow-derived macrophage.

### Comparison of miRNA-Modified Exosomes and Exosome Treatment Groups at 28 days Post Injury

**FIGURE 2:**
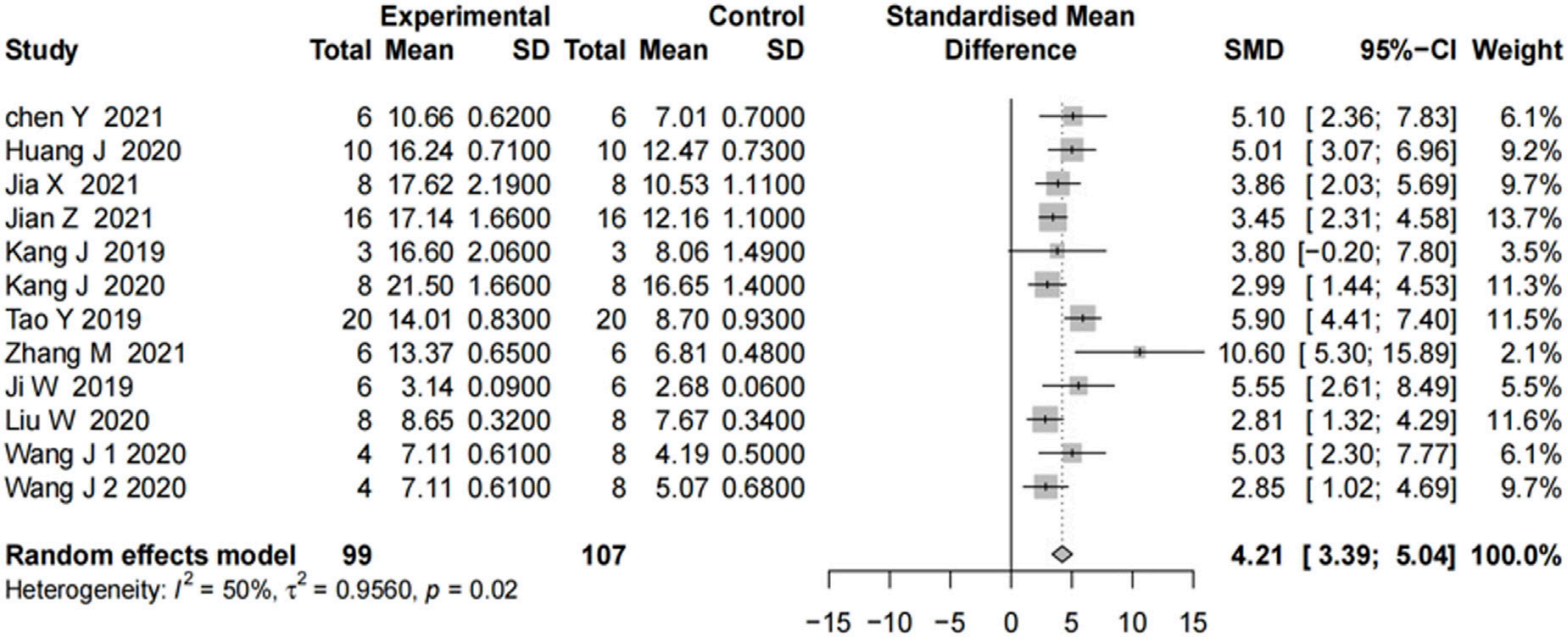
The forest plot shows the effect of MIR-modified exosome therapy on motor function on the 28^th^ days after spinal cord injury. A positive SMD represents an improvement in the sports score.

We analyzed 11 studies of motor function scores after SCI. Motor function scores at 28 days after the SCI event were measured in most studies. On day 28, all showed significant improvement in motor function scores in the groups treated with the miRNA-modified exosomes, compared with the groups treated with exosomes only. The results in Figure 2 showed moderate heterogeneity. To explore the source of heterogeneity, we conducted a subgroup analysis.

### Subgroup Analyses

We performed subgroup analyses for blinding, randomization, model, dose, and treatment duration (Table 2; Supplementary Figures S1-S5). The results of subgroup analysis revealed that there was no difference in the final effect among the different models. The treatment dose of 100 μg had a significant effect on the recovery of function after SCI. Overall, the mean effect of delayed treatment was more significant than immediate treatment in terms of motor function recovery. Jia (Jia et al., 2021) was not included in the subgroup analysis owing to treatment duration was unknown. The results, however, should be interpreted with caution because of the small sample size and the lack of direct evidence.

**TABLE 2:** Subgroup analysis of miRNA-modified exosomes therapies on locomotor score.

Subgroup	SMD, 95CI	I^2^ (%)		*p* value
Blinding
Yes(n = 6)	**4.64(3.66–5.63)**	38	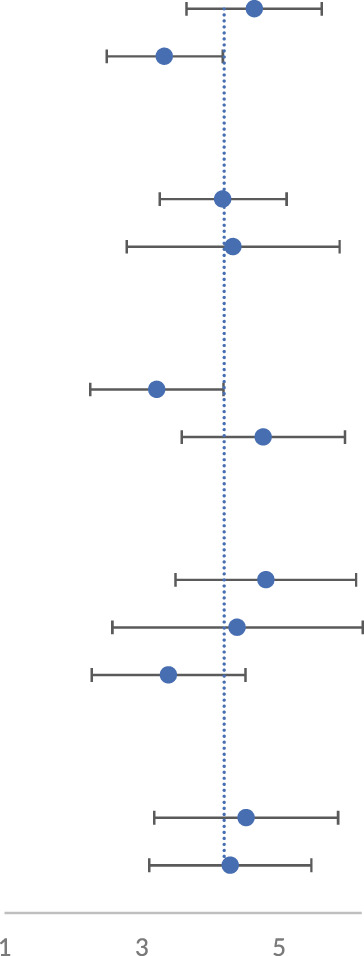	0.15
No(n = 6)	**3.33(2.48–4.17)**	48		0.08
Random			
Yes(n = 10)	**4.18(3.25–5.10)**	57		0.01
No(n = 2)	**4.33(2.78–5.88)**	0		0.34
Time			
Immediate(n = 4)	**3.22(2.25–4.19)**	0		0.52
Delay(n = 7)	**4.77(3.58–5.96)**	62		0.02
Dose			
100 μg(n = 4)	**4.81(3.50–6.13)**	60		0.06
200 μg(n = 5)	**4.39(2.56–6.21)**	63		0.03
Other(n = 3)	**3.39(2.27–4.51)**	0		0.75
Model			
Compression(n = 3)	**4.52(3.18–5.86)**	0		0.56
Contusion(n = 8)	**4.29(3.11–5.47)**	64		<0.01

### Quality Assessment

**FIGURE 3:**
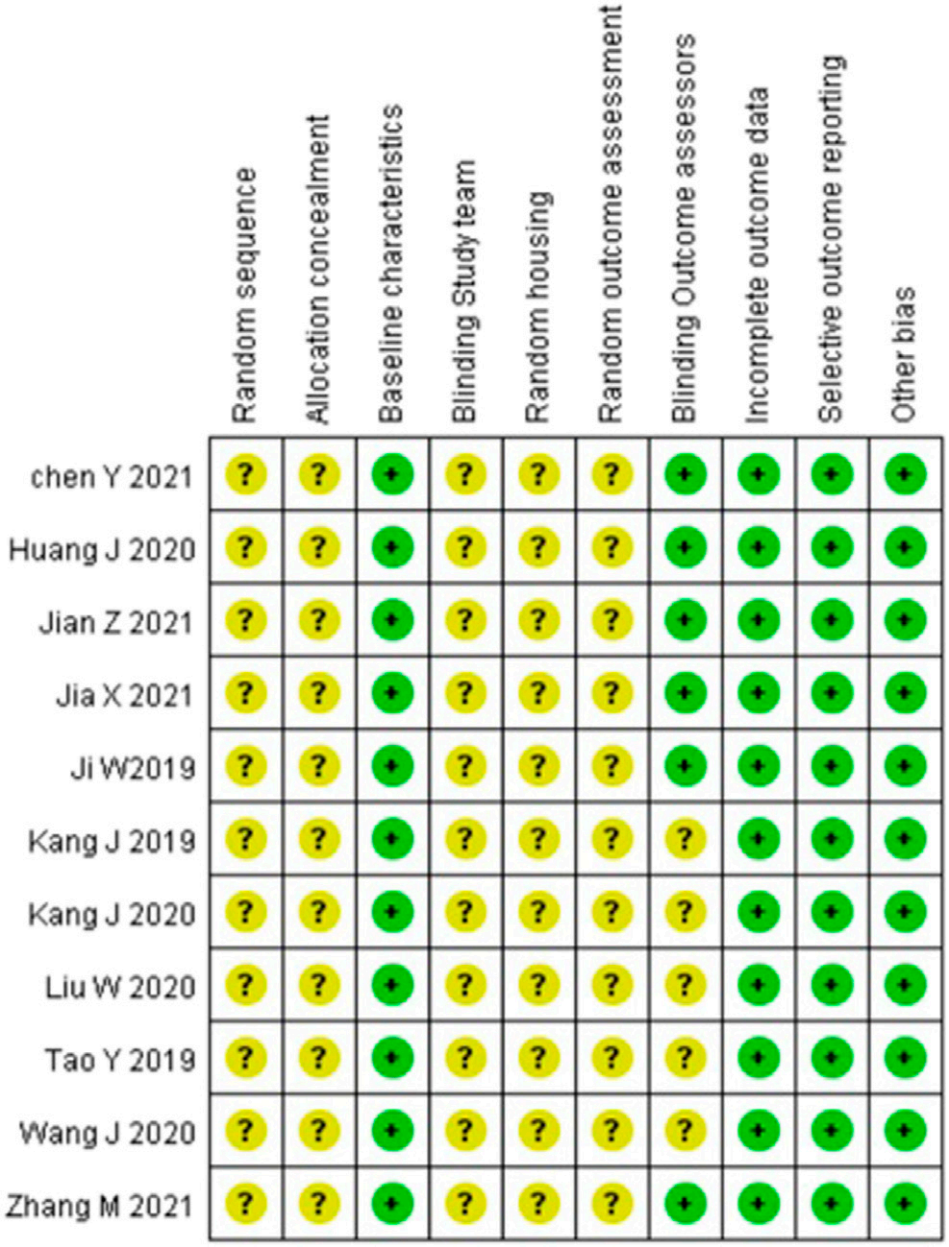
Article quality assessment using SYRCLE’s tool.

Article quality was assessed using SYRCLE’s ROB tool. Most articles performed both randomization and blinding, while a few performed either randomization or blinding. Most articles did not describe allocation concealment. Further details of the study quality score are presented in Figure 3.

**FIGURE 4:**
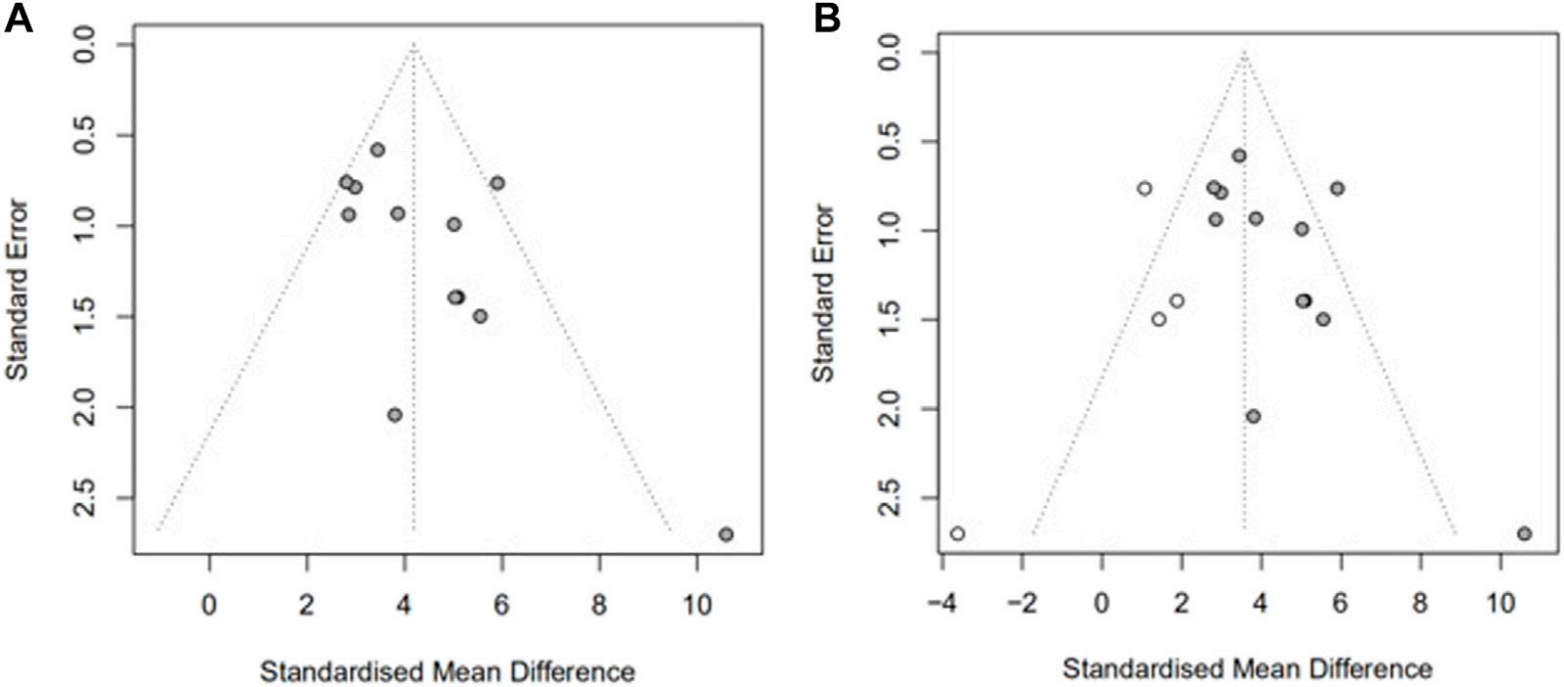
Assessment of publication bias in the locomotor score data. Funnel plots show pronounced asymmetry in locomotor score **(A)**. Trim-and-fill analysis of the locomotor score **(B)**, predicted 4 “missing” studies (unfilled circles).

We then assessed publication bias. As shown in Figure 4A, there was a significant funnel plot asymmetry in the exercise score data, indicating publication bias. Potential publication bias of primary outcomes was tested using Egger’s test *p* = 0.0390) (Hayashino et al., 2005). Trim-and-fill analysis (Figure 4B) estimated there were four “missing” unpublished studies on the left-hand side of the funnel plot. To identify the source of bias, we performed a subgroup analysis as above.

We summarized the clinical characteristics of spinal cord injury, including cause of injury and level of spinal cord injury, time point of treatment, administration of postoperative antibiotics, recovery of bladder function, and behavioral recovery (Table 3). We found high clinical correlation between most of the study models, but the use of postoperative antibiotics and recovery of bladder function were not described in some studies.

**TABLE 3:** Evaluation of the relevance of clinical characteristics of spinal cord injury.

Clinical characteristics	Chen Y 2021	Huang J 2020	Ji W 2019	Jia X 2021	Jiang Z 2021	Kang J 2019	Kang J 2020	Liu W 2020	Wang J 2020	Tao Y 2019	Zhang M 2021
Cause of injury: indirect trauma resulting in compression or contusion	+	+	+	+	?	+	+	+	+	+	+
Common injury level: the thoracic medulla	+	+	+	+	+	+	+	+	+	+	+
Time point for treatment is usually not immediately after the injury	−	+	+	?	+	−	−	−	+	+	+
Postoperative antibiotic treatment was given	?	?	+	?	?	?	?	?	+	+	?
Recovery of bladder function after surgery	?	?	+	?	?	?	?	+	+	?	+
outcome measure: orchestrates locomotion	+	+	+	+	+	+	+	+	+	+	+

(+) eligible (−) not eligible (?) unclear.

## Discussion

In this meta-analysis of the efficacy of miRNA-modified exosome treatment in animal models of SCI, we included 11 articles that met our inclusion criteria. In terms of the overall effect, miRNA-modified exosome therapy improved motor functional scores, compared with control or exosome-only therapy. We assessed ROB using SYRCLE’s tool and plotted funnel plots, and identified an estimated four unpublished motor score datasets by trim-and-fill analysis.

Currently, there is no definitive conclusion as to what is best for a single, multiple dose or continuous infusion of exosomes. Almost all studies included in the literature started acute treatment within 24 h of injury. Most studies used exosome doses of 100 and 200µg, and our results suggested that exosome treatment at a dose of 100 µg may be better for motor function repair. The main routes of drug administration for spinal cord injury include caudal vein injection, intrathecal injection, etc. Since intrathecal injection may further aggravate spinal cord injury, most studies have used the simple and easy-to-use caudal vein injection method. To further accurately deliver exosomes to the area of spinal cord injury, some scholars have investigated a number of drug delivery systems. Zhang (Zhang L. et al., 2021) designed paclitaxel-delivered exosome-collagen scaffolds via biospecific peptides to effectively retain exosomes in the scaffold for repair of spinal cord injury. Li (Li et al., 2020) immobilized human MSC-derived exosome grafts in a viscous hydrogel to treat spinal cord injuries. All these methods provide references for the clinical transformation of exosome therapy.

Most of the cellular sources of exosomes in the literature were MSCs. MSCs are considered a good source of exosomes because of their pluripotency, strong differentiation ability, and low ethical controversy (Liau et al., 2020). Because of their key role in intercellular communication, exosomes have been widely used as carriers of endogenous or exogenous cargo for the treatment of various diseases (Shen et al., 2018). Li (Li S.-P. et al., 2018) found that rats that received miR-133-modified exosomes recovered well, and that these exosomes promoted axon growth and reduced the levels of RhoA protein in the injured spinal cord, indicating that RhoA is a direct target of miR-133, and suggesting that the RhoA/ROCK signaling pathway plays a critical role in spinal cord neuron death after acute SCI (Wu et al., 2017). Chen found that exosomes derived from miR-26a-modified MSCs activate the PTEN-AKT-mTOR pathway, and promote axon regeneration and neurogenesis, and reduce glial scar formation in SCI(Chen et al., 2021). Our current meta-analysis findings suggest that miRNA-modified exosomes can significantly improve motor function in the rodent SCI model.

This study has the following limitations: (1) The data of the original study was limited. Important data such as drug dose, administration method, and method of model production were occasionally missing. In addition, some studies described only the volume or concentration of exosomes, and one article did not report the time of exosome injection. (2) The quality of the included studies varied. Most studies did not clearly describe the randomization design or allocation concealment. (3) The scoring criteria for motor scores differed, with BBB scores (ranging from 0 to 21), which are mostly used for motor functional evaluation in rats, and BMS scores (ranging from 0 to 9), which are mostly used for motor functional evaluation in mice. Furthermore, the observations were mostly subjective, and therefore, conclusions should be derived at with caution. We recommend the use of more objective tools, such as hindlimb strength and motor evoked potentials (MEP). (4) There is no international consensus on the establishment of a model for SCI. While most studies used the contusion model, others used other models, such as the compression model. Therefore, we suggest a standardized model for SCI. In addition, the frequency of exosome injection varied, although almost all studies started treatment within 24 h after the injury and were done by tail vein injection. We found that most studies reported positive results. Our meta-analysis showed publication bias in motor function results, which may have caused the effect size to be overestimated.

## Conclusion

The present meta-analysis suggests that the transplantation of miRNA-modified exosomes has a better effect on the recovery of motor function than exosome therapy alone in SCI. Additionally, model, dosage and time of administration are potential factors that affect the therapeutic effect. However, it remains unclear whether miRNA-modified exosomes are better than miRNA therapy alone in the treatment of SCI. While this meta-analysis provides a direction for future research, the mechanisms underlying the therapeutic action of miRNA-modified exosomes still need to be clarified.

## Supplementary Material

Supplementary Figure S1 Forest plot summarizing the relationship between blinding and motor function scores in preclinical models of SCI.

Supplemental Figure S2 Forest plot summarizing the relationship between random and motor function scores in preclinical models of SCI.

Supplementary Figure S3 Forest plot summarizing the relationship between timing of treatment and motor function scores in preclinical models of SCI.

Supplementary Figure S4 Forest plot summarizing the relationship between dose of treatment and motor function scores in preclinical models of SCI.

Supplementary Figure S5 Forest plot summarizing the relationship between animal model and motor function scores in preclinical models of SCI.

Supplementary Table S1 PRISMA 2009 Checklist.

Supplementary Table S2 Detailed search strategy.

Supplementary Table S3 Raw data file.

## Abbreviations

BBB, Basso, Beattie and Bresnahan; BMS, Basso Mouse Scale; MSCs, Mesenchymal stem cells; ROB, Risk of bias; SYRCLE, Systematic Review Centre for Laboratory Animal Experimentation; SCI, Spinal cord injury; SMD, standardized mean difference.
