# Supplementary figures and images for: The Effect of miRNA-Modified Exosomes in Animal Models of Spinal Cord Injury: A meta-Analysis
Source: Front Bioeng Biotechnol. 2022 Jan 6;9:819651. doi: 10.3389/fbioe.2021.819651 (PMC8770826; doi:10.3389/fbioe.2021.819651)

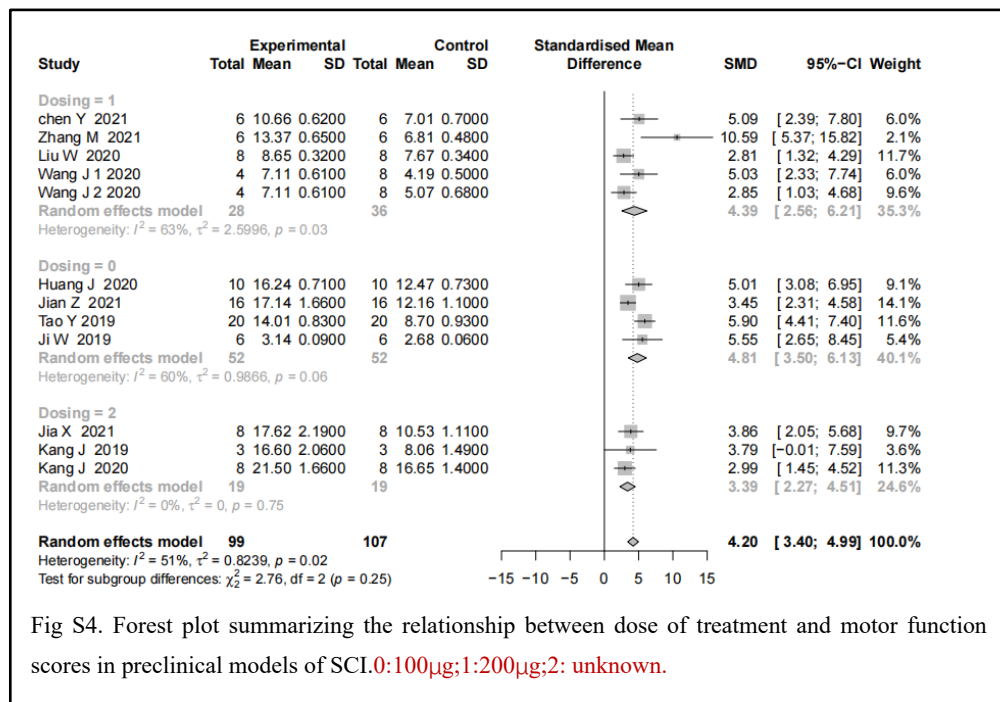

Supplement: Supplementary file 3 [file Image4.pdf]
