# Supplementary material for: The Effect of miRNA-Modified Exosomes in Animal Models of Spinal Cord Injury: A meta-Analysis
Source: Front Bioeng Biotechnol. 2022 Jan 6;9:819651. doi: 10.3389/fbioe.2021.819651 (PMC8770826; doi:10.3389/fbioe.2021.819651)
Supplement: Supplementary file 5 [file Image3.pdf]

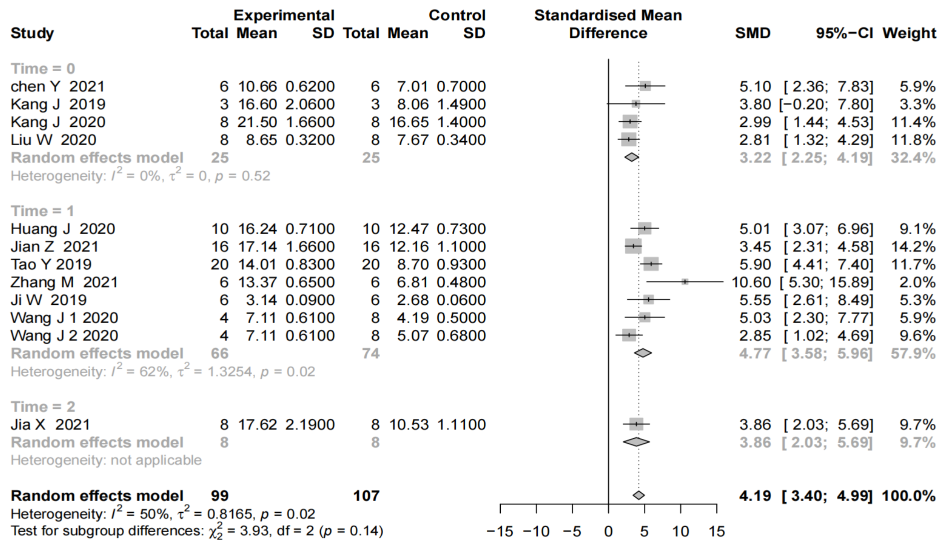

Fig S3. Forest plot summarizing the relationship between timing of treatment and motor function scores in preclinical models of SCI.0: immediate treatment ;1: delayed treatment ;2: unknown treatment duration.
