# Supplementary material for: The Effect of miRNA-Modified Exosomes in Animal Models of Spinal Cord Injury: A meta-Analysis
Source: Front Bioeng Biotechnol. 2022 Jan 6;9:819651. doi: 10.3389/fbioe.2021.819651 (PMC8770826; doi:10.3389/fbioe.2021.819651)
Supplement: Supplementary file 7 [file Table2.docx]

**Table S2. The detailed search strategy**

| **Electronic databases** | **Search** | **Search strategy** |  | **Results** |
| --- | --- | --- | --- | --- |
| **PUBMED** | #1 | (((((("Exosomes"[Mesh]) OR (Endosomes[Title/Abstract])) OR (Secretory Vesicles[Title/Abstract])) OR (Cell-Derived Microparticles[Title/Abstract])) OR (Exosome Multienzyme Ribonuclease Complex[Title/Abstract])) AND ((((((((((((((((((("MicroRNAs"[Mesh]) OR (MicroRNA[Title/Abstract])) OR (miRNAs[Title/Abstract])) OR (Micro RNA[Title/Abstract])) OR (RNA, Micro[Title/Abstract])) OR (miRNA[Title/Abstract])) OR (Primary MicroRNA[Title/Abstract])) OR (MicroRNA, Primary[Title/Abstract])) OR (Primary miRNA[Title/Abstract])) OR (miRNA, Primary[Title/Abstract])) OR (pri-miRNA[Title/Abstract])) OR (pri miRNA[Title/Abstract])) OR (RNA, Small Temporal[Title/Abstract])) OR (Temporal RNA, Small[Title/Abstract])) OR (stRNA[Title/Abstract])) OR (Small Temporal RNA[Title/Abstract])) OR (pre-miRNA[Title/Abstract])) OR (pre miRNA[Title/Abstract])) OR (miR[Title/Abstract]))) AND (((((((((((((((((((("Spinal Cord Injuries"[Mesh]) OR (Spinal Cord Trauma[Title/Abstract])) OR (Cord Trauma, Spinal[Title/Abstract])) OR (Trauma, Spinal Cord[Title/Abstract])) OR (Myelopathy, Traumatic[Title/Abstract])) OR (Traumatic Myelopathies[Title/Abstract])) OR (Injuries, Spinal Cord[Title/Abstract])) OR (Cord Injuries, Spinal[Title/Abstract])) OR (Spinal Cord Transection[Title/Abstract])) OR (Cord Transection, Spinal[Title/Abstract])) OR (Transection, Spinal Cord[Title/Abstract])) OR (Spinal Cord Laceration[Title/Abstract])) OR (Cord Laceration, Spinal[Title/Abstract])) OR (Laceration, Spinal Cord[Title/Abstract])) OR (Post-Traumatic Myelopathy[Title/Abstract])) OR (Myelopathies, Post-Traumatic[Title/Abstract])) OR (Post Traumatic Myelopathy[Title/Abstract])) OR (Spinal Cord Contusion[Title/Abstract])) OR (Contusion, Spinal Cord[Title/Abstract])) OR (Cord Contusion, Spinal[Title/Abstract])) |  | 20 |
|  |  |  |  |  |
| **EMBASE** | #1 | ' spinal cord injury '/exp |  | 85010 |
|  | #2  #3 | ' microrna '/exp  ' exosome '/exp |  | 189336  37835 |
|  | #4 | #1 AND #2 AND #3 |  | 61 |
|  |  |  |  |  |
| **Web of Science** | #1 | TS=(Exosomes) OR TS=(Endosomes) OR TS=(Secretory Vesicles) OR TS=(Cell-Derived Microparticles) OR TS=(Exosome Multienzyme Ribonuclease Complex) |  | 72127 |
|  | #2 | TS=(MicroRNAs) OR TS=(miRNAs) OR TS=(Micro RNA) OR TS=(RNA, Micro) OR TS=(Primary MicroRNA) OR TS=(MicroRNA, Primary) OR TS=(Primary miRNA) OR TS=(pri-miRNA) OR TS=(RNA, Small Temporal) OR TS=(Temporal RNA, Small) OR TS=(stRNA) OR TS=(Small Temporal RNA) OR TS=(pre-miRNA) OR TS=(miR) |  | 224520 |
|  | #3 | TS=(Spinal Cord Injuries) OR TS=(Spinal Cord Trauma) OR TS=(Cord Trauma, Spinal) OR TS=(Trauma, Spinal Cord) OR TS=(Myelopathy, Traumatic) OR TS=(Traumatic Myelopathies) OR TS=(Injuries, Spinal Cord) OR TS=(Cord Injuries, Spinal) OR TS=(Spinal Cord Transection) OR TS=(Cord Transection, Spinal) OR TS=(Transection, Spinal Cord) OR TS=(Spinal Cord Laceration) OR TS=(Cord Laceration, Spinal) OR TS=(Laceration, Spinal Cord) OR TS=(Post-Traumatic Myelopathy) OR TS=(Myelopathies, Post-Traumatic) OR TS=(Post Traumatic Myelopathy) OR TS=(Spinal Cord Contusion) OR TS=(Contusion, Spinal Cord) OR TS=(Cord Contusion, Spinal) |  | 122173 |
|  | #4 | #1 AND #2 AND #3 |  | 72 |
